# P172: Innovate and educate

**DOI:** 10.1186/2047-2994-2-S1-P172

**Published:** 2013-06-20

**Authors:** M Lin

**Affiliations:** 1 Singapore General Hospital, Singapore, Singapore

## Introduction

One of the lessons we learnt post-SARS is the value of an informed healthcare workforce on the importance of infection control and their competence in its practices. Like many, we instituted mandatory orientation in Infection Prevention and Control (IPC) since then, for the 9,000 healthcare workers at our 1600-bedded acute tertiary care hospital. However, we face challenges in having the new hires trained early enough in their job for their safety.

## Methods

An attendance rate for the ICT is set at a goal >90% new staff completing the orientation within 1 month of hire. With help from Human Resource, reminders were sent on monthly basis since June 2012 to no-show attendees to their respective managers to attend the next available session. Secondly, we introduced blended learning, education that combines face-to-face classroom methods with computer-mediated activities, in an attempt to enhance our teaching. E-orientation modules on essential infection control principles and practices were developed and piloted first amongst medical students in June 2012. E-competency modules are developed for ICU staffs to help assess their knowledge on the VAP, CLABSI and CAUTI bundles. These staffs are given a year to complete these modules and their assessment scores are tracked.

## Results

We are able to achieve an improvement in attendance rate at orientation from an average of 60% to 80% in year 2012. The e-orientation pilot was a success and hence, the plan is now for it to be next rolled out hospital-wide.

## Conclusion

Today’s workforce will see an increasing number of Generation Y whom are techno savvy and less inclined to sit through a lecture. Innovative use of technology can enhance staff learning and education in IPC in these staffs. It is critical to have a workforce that is knowledgeable of IPC practice. It is equally important that their competency in IPC be assessed regularly to ensure safe practices.

## Disclosure of interest

None declared

